# The Relationship between Clinical Environment and Adverse Events Reporting: Evidence from Lithuania

**DOI:** 10.3390/healthcare12020252

**Published:** 2024-01-19

**Authors:** Juste Kiviliene, Renata Paukstaitiene, Alessandro Stievano, Aurelija Blazeviciene

**Affiliations:** 1 Department of Nursing, Faculty of Nursing, Lithuanian University of Health Sciences, 44307 Kaunas, Lithuania; aurelija.blazeviciene@lsmu.lt; 2 Department of Physics, Mathematics, and Biophysics, Medical Academy, Lithuanian University of Health Sciences, 44307 Kaunas, Lithuania; renata.paukstaitiene@lsmu.lt; 3 Department of Clinical and Experimental Medicine, University of Messina, 98100 Messina, Italy; astievano@unime.it

**Keywords:** clinical environment, adverse events, patient safety, nursing practice

## Abstract

Background: The clinical environment plays a crucial role in patient safety, as it encompasses the physical, organizational, and cultural aspects of healthcare delivery. Adverse events, such as active errors, can often be attributed to systemic issues within the clinical environment. Addressing and improving environmental factors is essential for minimizing adverse events and enhancing overall patient care quality. Methods: A descriptive, cross-sectional design was applied. The study utilized two questionnaires: the Reporting of Clinical Adverse Events Scale (RoCAES) and the Revised Professional Practice Environment (RPPE) scale. A total of 1388 questionnaires were fully filled out, with a response rate of 71 percent. Results: Nurses who expressed higher levels of satisfaction with various aspects of the clinical environment were more inclined to indicate their intention to report adverse events in the future. These positive relationships suggest that a contented clinical environment fosters a greater willingness among nurses to report adverse event occurrences. Conclusion: The findings of our study support the evidence that demonstrated that the clinical environment plays a significant role in influencing the reporting of adverse events in healthcare settings. It significantly influences nurses’ attitudes, quality of care, and adverse event reporting rate.

## 1. Introduction

The clinical environment plays a crucial role in ensuring patient safety. It encompasses various factors that can directly impact the well-being of patients during their healthcare experience. Patient safety stands as a vital priority within the hospital setting [1,2], and it is profoundly influenced by nursing care, which further affects clinical outcomes, patient satisfaction with the care they receive, and the overall satisfaction of nursing staff with the care they provide [3]. Several factors can significantly influence the clinical environment and, in turn, have a direct impact on patient safety. In the context of healthcare delivery, front-line providers can make active errors, such as administering the wrong medication to a patient [4]. However, it is important to recognize that these errors often arise due to contributing factors, known as latent conditions, which are frequently related to the design of the physical environment. These latent conditions can manifest as noisy, cluttered, and crowded patient rooms, which often go unnoticed in the healthcare delivery system [4]. Unfortunately, patients and staff members bear the consequences of such conditions, leading to adverse events such as errors, falls, or healthcare-associated infections. For instance, inadequate placement of sinks for handwashing can result in low compliance with hand hygiene requirements, subsequently leading to an increased risk of healthcare-associated infections [4]. Another important factor is staffing and the workforce. Evidence supports the notion that organizational features of nursing, such as improved staffing ratios, are strongly associated with positive patient outcomes [5,6]. Enhanced staffing ratios have been linked to reduced mortality rates, fewer falls, and lower infection rates among patients [6]. It is crucial for healthcare managers to invest in nursing staff, recognizing them as fundamental contributors to the promotion of patient safety [5,6]. By highlighting the importance of collaboration and encouraging smooth transfers of care, the risk of errors and adverse events is significantly reduced [7].

Clear policies and procedures also play a crucial role in guiding healthcare providers to deliver safe and standardized care. By establishing evidence-based policies and procedures, healthcare organizations can promote the use of the latest research and best practices in patient care, ultimately improving the quality of care and patient outcomes [8].

Creating a safer and more effective healthcare environment that benefits both healthcare providers and patients also requires the successful integration of technology into healthcare, which depends on the attitudes and experience of nurses as well as ongoing education and training appropriate to their technological skills [9,10]. The clinical environment and the educational background of nurses significantly influence both the incidence of adverse events and the subsequent reporting of these occurrences.

Studies conducted by Spanish and German researchers state that nurses often do not report adverse events that occur in the hospital [11,12]. The most important barriers are lack of support from managers and other staff, fear of consequences and punishment, too much work, education, professional duties, doubts about whether it is necessary and what needs to be reported, being afraid to appear as an incompetent specialist, seeing no point in reporting, and many more [11,12,13,14,15]. These findings suggest that nursing team members should be more knowledgeable about adverse events. Therefore, through various tactics, such as encouraging the reporting of adverse events and organizing training sessions for nurses, managers might offer the necessary conditions to enhance patient safety culture and decrease adverse occurrences [16,17,18,19]. This all includes creating and ensuring a safe clinical environment.

The aim of this study was to identify the relationship between clinical environmental and adverse event reporting in university and municipality-level hospitals in Lithuania.

Through this study, we intend to delve deeper into the essential role of the work environment in healthcare, emphasizing its impact on the provision of safe and quality care and its influence on the retention of healthcare professionals in the system. The results of our study could contribute to existing evidence, highlighting the significant impact of the clinical environment on the reporting of adverse events in healthcare institutions. Additionally, it can help uncover the personnel needs for the development of new competencies.

## 2. Materials and Methods

A descriptive, cross-sectional design was applied in this study.

### 2.1. Settings and Sample

Registered nurses were recruited from one university hospital, which is the largest healthcare institution in Lithuania, and from seven large municipality hospitals representing the seven administrative regions in Lithuania (Vilnius, Kaunas, Klaipėda, Panevėžys, Šiauliai, Alytus, and Marijampolė).

The university hospital has 2201 beds and 39 departments devoted to all medical and surgical specialties, providing the highest quality healthcare to patients from all over the country and abroad. Municipal hospitals are multi-profile hospitals that also provide both general and specialized healthcare services and have a combined total of 4552 beds. The collective bed capacity across the university and municipal hospitals amounts to 6753 beds.

In the survey, we included all nurses providing general and specialized healthcare services to hospitalized patients in municipal and university hospitals.

Ethical approval was provided by the Regional Biomedical Research Ethics Committee (permit number Nr. BE-2-14).

### 2.2. Translation Procedure

With the consent of the authors, linguistic adaptation processes were carried out in both questionnaires. This process involved forward translation, where the original questionnaires were translated from the source language (English) to the target language (Lithuanian) by one of the authors. Subsequently, back translation was conducted by another co-author who is proficient in both the source and target languages. In terms of substantive content, no significant differences were observed between the original instruments and the English versions of the questionnaires. Validation of the applied measures included testing with a specially selected group of relevant participants, nurses. The purpose of this testing was to evaluate alternative wordings, to understand comprehensibility, to clarify the nuances of interpretation, and to ensure the cultural suitability of the translation. According to the general assessment and decision of all authors, the questionnaires were recognized as suitable for use in the context of Lithuania.

### 2.3. Measures

To identify clinical, environmental, and adverse events, two questionnaires were used. Nurses’ attitudes toward adverse events were measured by the Reporting of Clinical Adverse Events Scale (RoCAES). The questionnaire was originally developed and validated in the UK by Wilson, Bekker, and Fylan in 2008 [20]. It consists of 25 questions and assesses five domains related to staff attitudes towards adverse events: perceived blame and criteria for identifying events that should be reported; perceptions of colleagues’ expectations; perceived benefits of reporting; and clarity of reporting procedures. The Cronbach’s alpha of the questionnaire used in our study was equal to 0.78.

The other questionnaire concerned the assessment of the nurses’ professional practice environment using the Revised Professional Practice Environment (RPPE) scale. This inquiry form was formulated on the foundation of the Professional Practice model. This model was developed through an inductive process at the Massachusetts General Hospital in Boston. Its purpose was to establish a common vision for six clinical disciplines and gain a holistic comprehension of the elements that govern professional practice within their hospital [21]. The scale consists of 42 questions and encompasses eight distinct sub-scales, each focusing on specific aspects like leadership, autonomy and control over clinical practice, communication about patients, teamwork, handling disagreements, staff relationships, internal work motivation, and cultural sensitivity. Cronbach’s alpha for this questionnaire used in our study was 0.87.

In both questionnaires, nurses were asked to select the most appropriate response on a 4-point Likert scale (1 = strongly disagree, 2 = disagree, 3 = agree, 4 = strongly agree).

Demographics collected included age, education, current workplace, and work tenure.

### 2.4. Data Collection

The study provides a comprehensive overview of the nursing landscape in Lithuania, drawing data from the Department of Statistics. In 2021, there were 22,079 practicing nurses in the country. The research focused on seven major cities, including Vilnius, Kaunas, Klaipėda, Šiauliai, Panevėžys, Alytus, and Marijampolė, where 6767 nurses were employed. The sample size for the study was determined using a 5 percent margin of error and a 95 percent probability level. Although the calculated sample size was 364 nurses, the decision was made to include all nurses working in the study hospitals. The inclusion criteria for participants comprised holding a nurse position, working in an adult inpatient unit, and providing consent to participate.

The survey was conducted between October 2021 and January 2022. The questionnaire and the consent form to participate in the study were delivered to the nurses personally at their workplace. One week was allotted for filling out and returning the questionnaire. To increase respondent trust and avoid inadvertent identification, participants submitted completed questionnaires in sealed envelopes directly to the researcher. It is necessary to emphasize that even the researcher did not know about the identities of the research participants. Out of the 1955 surveys distributed, 1388 questionnaires were fully filled out, with a response rate of 71 percent. Depending on the hospital, the response rate ranges from 50 to 92 percent.

### 2.5. Data Analysis

The survey data underwent analysis using the statistical software package SPSS for Windows 29.0 (SPSS Statistics for Windows). Mean and standard deviation (SD) were employed to describe normally distributed quantitative variables, while the median, minimum, and maximum values were used for non-normally distributed quantitative or ranked variables. For qualitative variables, frequency and relative frequency (%) were utilized. The Chi-square test for independence (homogeneity) compared two categorical variables. Observed correlations and differences were considered statistically significant if the *p*-value was below 0.05.

Exploratory factor analysis was conducted on the questions from both the RPPE and RoCAES questionnaires. Principal components with orthogonal Varimax rotation were used for factor extraction. The number of extracted factors corresponded to the number of principal components with a true value greater than 1. Bartlett’s sphericity criterion *p*-value < 0.001 for both questionnaires and the Kaiser-Meyer-Olkin index KMO = 0.871 for the RPPE questionnaire and KMO = 0.848 for the RoCAES questionnaire indicated that the data contained statistically significantly correlated variables and were sufficiently suitable for factor analysis.

Also, we computed factor value estimates as the meaning of the question values within each factor. Using these averages, nurses were divided into two groups based on a cutoff point of 2.5. Scores below 2.5 were classified as the ‘disagreeing’ or ‘dissatisfied’ group (negative answer), while scores equal to or exceeding 2.5 were categorized as the ‘agreeing’ or ‘satisfied’ group (positive answer).

## 3. Results

Table 1 describes the characteristics of the sample by hospital where the nurses worked. The nurse participants’ ages ranged from 22 to 76, with an average age of 47.21 (SD = 11.48) years, and their working experience ranged from 1 to 50 years, with an average age of 24.12 (SD = 13.28) years. More than twenty percent of nurses had higher university education—197 (14.3%) had a university bachelor’s degree and 84 (6.1%) had a master’s degree.

### 3.1. Nurses’ Attitude to Clinical Practice Environment

When analyzing nurses’ attitudes toward the professional environment, the research findings indicated that nurses rated the “Workplace Internal Motivation” domain most positively, with an average score of 3.27. The components most highly valued by respondents were the sense of great responsibility for their work and the satisfaction derived from performing their job well; both received an average rating of 3.57.

The second domain that garnered a rating above 3 was “Patient-related Communication,” with the highest regard given to the ability to access all necessary patient information when needed (mean score: 3.03).

Conversely, the lowest scores were attributed to the “Control of the Practice” domain, with an average rating of 2.57. Among the components within this domain, the statement “My department has enough nurses to ensure quality patient care” received the lowest rating, averaging 2.36 (Table 2).

### 3.2. Nurses’ Attitude to Adverse Events

The research findings indicate that nurses hold a favorable view of the hospital’s adverse event reporting system, with a mean rating of 2.87. They expressed that the hospital where they are employed has well-defined, standardized procedures for reporting adverse events, receiving a mean rating of 2.88. However, respondents’ assessments were less positive in the context of “Promoting a Supportive Culture for Adverse Event Reporting”, with a mean rating of 2.54.

Nurses’ evaluations were less favorable in the domain of “Challenges in Reporting Adverse Events”, where they indicated that they are not permitted to report adverse events (mean 1.94) and expressed the belief that their colleagues are not concerned when adverse events occur (mean 2.06) (Table 3).

### 3.3. Relationship between Clinical Environment and Adverse Events Reporting

Upon conducting an extensive analysis of the relationships, we discovered statistically significant differences in one group. Specifically, among nurses who expressed satisfaction with their work as a team, they did not report the occurrence of adverse events more often (72.7%). The remaining factors within the clinical environment showed no significant influence on the decision to report or not report adverse events (Table 4).

During the assessment of the relationship between nurses’ work environment and their intention to report adverse events in the future, we observed significant relationships in nearly all groups. Nurses who expressed higher levels of satisfaction with various aspects of the clinical environment, such as leadership and autonomy (89.0%), general relations and communication (86.4%), teamwork (73.3%), disagreement and conflict resolution (77.9%), and internal work motivation (97.9%), were more inclined to indicate their intention to report adverse events in the future. These positive relationships suggest that a contented clinical environment fosters a greater willingness among nurses to report adverse event occurrences (Table 5).

## 4. Discussion

The work environment holds significant importance in ensuring the provision of safe and high-quality care, as well as in retaining healthcare professionals within the healthcare system. The study reveals that nurses predominantly view the “Workplace Internal Motivation” and “Patient-related Communication” domains positively, emphasizing a strong sense of responsibility and satisfaction derived from job performance. However, concerns arise in the “Control of the Practice” domain, particularly regarding nurse staffing for ensuring quality patient care. While nurses generally favor the hospital’s adverse event reporting system, challenges exist in promoting a supportive culture for reporting adverse events. Remarkably, nurses satisfied with teamwork are less likely to report adverse events, while overall satisfaction in various aspects of the clinical environment positively influences the intention to report adverse events in the future. The findings from our study concerning nurses’ attitudes towards the professional practice environment align with outcomes from research conducted by other researchers. Maintaining patient safety is closely tied to having sufficient staffing levels and a workforce with the appropriate skills. Internal work motivation—the drive and dedication that employees have towards their jobs—plays a significant role in ensuring that patient safety remains a top priority [22]. Notably, internal work motivation is most important for all nurses and has consistently garnered the highest proportion of positive responses in our and other studies. In other words, the majority of nurses believe that their self-perception improves when they work in their departments. They experience a strong sense of responsibility for their professional tasks as well as satisfaction when they perform their duties well [23,24,25,26,27]. Meanwhile, our respondents reported being quite satisfied with the control of their practice, but in contrast to our study, researchers using the same instrument found that nurses tended to rate their satisfaction with the control of professional practice worse than the remaining factors [24,26,28]. Nonetheless, all respondents concur that there is indeed a shortage of nurses in this field. According to nurses, the organization of nursing tasks in the department is insufficient, so there is not enough time to communicate with the patients. In addition, there is not enough time and opportunities for cooperation between nurses on patient care issues [24,25,26,27,28].

Effective communication and teamwork are also critical for patient safety. However, nurses expressed less favorable evaluations regarding their teamwork experiences. Specifically, about the insufficiency of cooperation from staff in other departments when required. Furthermore, a perception emerged that the staff in other departments held unfavorable opinions about another department. Additionally, inadequate working relationships with hospital staff were identified as contributing factors that curtailed work efficiency within their department [23,29]. Special emphasis should be placed on recognizing the significance of teamwork. Teamwork is an essential element of ensuring patient safety, and there is a strong relationship between effective teamwork and better reporting of adverse events [30,31]. However, our study revealed the opposite results. According to our nurses’ assertions, individuals who expressed satisfaction with their teamwork experienced a lower frequency of reporting adverse events. This could be linked to the apprehension of harming one’s personal image or reputation within the healthcare team. This fear is often tied to the desire to maintain a sense of professionalism, trust, and friendship. Positive reinforcement could be recognition and appreciation of nurses who report adverse events, which could demonstrate that their contribution to patient safety is valued and respected.

Clear policies and procedures are necessary to guide healthcare providers in delivering safe and standardized care. Assessing nurses’ attitudes towards adverse events, the opinions of nurses differed between studies conducted in different countries due to policy and cultural differences and their understanding of professional roles. In Germany, the formal training of healthcare professionals does not encompass risk management and adverse event management. Consequently, workers have become accustomed to reporting processes, which unfortunately leads to a tendency to attribute blame [12]. In contrast, in England, clinical adverse event reporting constitutes a key component of the National Health Service’s clinical governance framework. It plays a crucial role in enhancing the safety and quality of services by affording staff the opportunity to glean insights from past errors and incidents, thereby facilitating a culture of continuous improvement. This results in a more neutral and professional approach, wherein the emphasis is on perceiving benefits rather than assigning blame [20]. In China, the principle of harmony has great cultural significance, and individuals who report adverse events involving colleagues may be socially isolated by others. Although establishing responsibility is recognized as one aspect of adverse event reporting, it is not the primary goal [32]. Nurses in our country claim that the hospitals where they work have well-defined procedures for reporting adverse events and for determining which adverse events should be reported. Additionally, they also indicated that they understand that although adverse events cannot be avoided, reporting them is necessary, and they noted that colleagues are concerned when adverse events occur. The respondents’ assessments regarding a favorable culture for reporting adverse events were less positive. They indicated a lack of encouragement from experienced colleagues and the hospital’s adverse event monitoring unit to report errors.

A positive safety culture in the clinical environment is also an extremely important aspect. This culture emphasizes reporting and learning from errors, near-misses, and adverse events. It encourages healthcare providers to speak up about safety concerns, engage in continuous quality improvement initiatives, and actively participate in patient safety training and education programs [7,33]. In evaluating the relationship between nurses’ work environment and their intention to report adverse events in the future, our findings are closely related to those of other researchers. Adverse events reporting was found to be associated with higher levels of nurse satisfaction in various aspects of the clinical domain. Nurses conveyed the most favorable aspects as manager support, leadership, and nurse-physician relations. Notably, the nurses’ perception of their work environment displayed a substantial link to the reporting of adverse events, with a more positive perception corresponding to a higher tendency to report such events [14,31,34,35]. In other words, a conducive and supportive work environment seems to encourage nurses to be open about reporting incidents that may have caused harm to patients.

It is essential to highlight the discrepancy between nurses not reporting adverse events currently while expressing an intention to do so in the future. The delay in reporting adverse events by nurses could be influenced by the culture within the healthcare organization, which may discourage reporting, making nurses hesitant to come forward with adverse events, or nurses might be apprehensive about the potential consequences of reporting adverse events. They may fear blame, disciplinary action, or a negative impact on their professional reputation. Addressing the organizational culture may be necessary to foster an environment that encourages transparency.

### Strengths and Limitations

A strength of our study is its innovative approach, being the first in the country to assess the relationship between the clinical environment and adverse events in nursing. Also, for the first time in our country, research instruments created and recognized by foreign authors were applied. Another strength of the study was the systematic data collection procedure, which was conducted at the same time in each hospital. The data can be considered representative considering that nurses working in a major municipal hospital and the largest university hospital in the country were approached to participate. The utilization of these assessment tools for evaluating the professional practice environment and reporting adverse events introduces a new perspective on comprehending our healthcare systems and promoting patient safety.

The limitations of the study should also be mentioned. The questionnaire examines nurses’ perceptions and attitudes, which may not precisely mirror actual work circumstances. Moreover, certain questions might have been interpreted differently by participants, possibly exerting an influence on the resultant outcomes. Furthermore, the application of factor analysis to the questionnaires revealed certain items that diverged from factor scales validated in other countries. These questions need further consideration.

## 5. Conclusions

The relationship between the clinical environment and adverse events is complex and multifaceted, requiring careful consideration and management. The findings of our study support the evidence that demonstrated that the clinical environment plays a significant role in influencing the reporting of adverse events in healthcare settings. It significantly influences nurses’ attitudes, quality of care, and adverse event reporting rate. A positive work environment correlates with a higher tendency to report such incidents, indicating that a supportive environment encourages openness. Additionally, nurses feel a strong sense of responsibility for their tasks and experience satisfaction when they perform them well. By recognizing the critical role of the clinical environment and implementing strategies to optimize it, healthcare organizations can significantly increase adverse event reporting rates and ensure the highest level of patient safety and quality of care. Addressing this issue may necessitate a transformation in the organizational culture of the institution. This transformation could involve empowering the organization and fostering a blame-free culture that promotes open communication. It is also very important to continue research in this area, conducting not only quantitative but also qualitative research and deepening knowledge by listening to the experiences and opinions of each nurse.

## Figures and Tables

**Table 1:** Demographic characteristics of nurses by hospital.

Characteristics	Vilnius N (%)	Kaunas N (%)	Klaipėda N (%)	Panevėžys N (%)	Šiauliai N (%)	Alytus N (%)	Marijampolė N (%)	University Hospital N (%)
Age (in years)								
≤35	13 (17.3)	33 (28.4)	26 (38.8)	48 (14.9)	31 (14.6)	3 (5.4)	3 (7.1)	94 (20.2)
36–50	42 (56.0)	31 (26.7)	14 (20.9)	125 (38.8)	85 (39.9)	11 (19.6)	12 (28.6)	173 (37.2)
>51	20 (26.7)	52 (44.8)	27 (40.3)	149 (46.3)	97 (45.5)	42 (75.0)	27 (64.3)	198 (42.6)
Education								
Medical school	20 (26.7)	54 (46.6)	25 (37.3)	154 (46.7)	98 (46.2)	46 (69.7)	28 (60.9)	204 (43.9)
Bachelor’s degree	52 (69.3)	52 (44.8)	40 (59.7)	170 (51.5)	109 (51.4)	18 (27.3)	17 (37.0)	205 (44.1)
Master’s degree	3 (4.0)	10 (8.6)	2 (3.0)	6 (1.8)	5 (2.4)	2 (3.0)	1 (2.2)	55 (11.8)
Work tenure								
0–5	10 (14.5)	29 (25.2)	25 (40.3)	37 (11.7)	26 (12.3)	1 (1.6)	3 (7.3)	79 (17.2)
6–15	10 (14.5)	12 (10.4)	4 (6.5)	50 (15.8)	27 (12.8)	3 (4.8)	1 (2.4)	63 (13.5)
16–25	16 (23.2)	15 (13.0)	5 (8.1)	53 (16.8)	40 (19.0)	8 (12.9)	4 (9.8)	96 (20.6)
26–31	21 (30.4)	17 (14.8)	8 (12.9)	54 (17.1)	42 (19.9)	8 (12.9)	12 (29.3)	68 (14.6)
>32	12 (17.4)	42 (36.5)	20 (32.3)	122 (38.6)	76 (36.0)	42 (67.7)	21 (51.2)	132 (32.9)

**Table 2:** Nurses’ attitude towards the professional practice environment domains.

Subscale and Items	Mean	SD	Median	Min–Max	Positive Answers N (%) *
**Clinical Practice: Leadership and Autonomy**	**2.99**	**0.47**	**3**	**1–4**	**1209 (87.1)**
Leadership supports nursing.	2.72	0.794	3	1–4	901 (64.9)
In my department, nurses control their activities.	3.03	0.614	3	1–4	1205 (86.8)
I can independently make important decisions related to patient care.	2.81	0.747	3	1–4	966 (69.6)
The head nurse of my department is a good manager and leader.	3.26	0.728	4	1–4	1208 (87.03)
My head nurse supports nurses in making decisions, even when there are conflicts with doctors.	3.13	0.738	3	1–4	1158 (83.4)
**Control of the Practice**	**2.57**	**0.63**	**2.6**	**1–4**	**732 (87.1)**
The work of nurses is well organized in the department, which allows more time to be spent with the patient.	2.75	0.772	3	1–4	899 (64.8)
I have enough time and opportunity to discuss patient care with other nurses.	2.63	0.773	3	1–4	813 (58.6)
My department has enough nurses to ensure quality patient care.	2.36	0.923	2	1–4	605 (43.6)
We have enough staff members to get the work done in the department.	2.43	0.87	2	1–4	614 (44.2)
There is an opportunity to work in a highly specialized patient care department.	2.67	0.792	3	1–4	839 (60.4)
**Patient-related Communication**	**3.03**	**0.57**	**3**	**1–4**	**1174 (84.6)**
Information about the patient’s condition is always available when I need it.	3.06	0.624	3	1–4	1196 (86.2)
I quickly receive information about changes in my patient’s condition.	3	0.649	3	1–4	1151 (82.9)
Information about patient care is transmitted immediately.	3.03	0.715	3	1–4	1118 (80.5)
**Work in a Team**	**2.76**	**0.58**	**2.67**	**1–4**	**982 (70.7)**
Staff of my department do not receive the necessary cooperation when needed from staff of other departments.	2.74	0.689	3	1–4	954 (68.7)
I think that the staff in another department has a bad opinion about my department.	2.85	0.799	3	1–4	996 (71.8)
Inadequate working relationships with other hospital staff limit work efficiency in my department.	2.68	0.788	3	1–4	852 (61.4)
**Dealing with Conflicts**	**2.74**	**0.5**	**2.83**	**1–4**	**1045 (75.3)**
Staff in my department avoid conflict.	2.75	0.69	3	1–4	949 (68.4)
In my department, the attitudes of all staff are well considered to find the best solution to the problem.	2.76	0.712	3	1–4	945 (68.1)
Everyone in my department works hard to find the best possible solution to the problem.	2.86	0.693	3	1–4	1025 (73.8)
In my department, all staff withdraw from the conflict and resolve it until everyone is satisfied with the decision.	2.59	0.727	3	1–4	778 (56.1)
Every member of staff in my department contributes to conflict resolution with their experience and knowledge.	2.69	0.664	3	1–4	891 (64.2)
The staff participating in the conflict resolved the dispute by consensus.	2.79	0.63	3	1–4	1029 (74.1)
**Workplace Internal Motivation**	**3.27**	**0.43**	**3.25**	**1–4**	**1347 (97.0)**
My opinion of myself is better when I work in my department.	2.97	0.648	3	1–4	1140 (82.1)
I feel bad when I realize that I do a task worse than I should.	3.1	0.666	3	1–4	1212 (87.3)
I feel a great responsibility for the work I do.	3.57	0.563	4	1–4	1358 (97.8)
I feel great satisfaction when I do my job well.	3.57	0.568	4	1–4	1350 (97.3)
I work in a demanding job that motivates me to work as best as I can.	3.36	0.645	3	1–4	1287 (92.7)
Working in my department gives me the opportunity to gain new knowledge and skills.	3.25	0.659	3	1–4	1249 (90.0)
I am motivated to work well because I am empowered by my work environment.	3.19	0.675	3	1–4	1213 (87.4)
Working in this environment increases my sense of professional growth.	3.16	0.68	3	1–4	1199 (86.4)

* Positive answers: for subscales—average value of all items ≥2.5; for separate items—sum of “agree” and “strongly agree” cases.

**Table 3:** Nurses’ attitude towards adverse events.

Subscale and Items	Mean	SD	Median	Min–Max	Positive Answers N (%) *
**Challenges in Reporting Adverse Events**	**2.04**	**0.562**	**2**	**1–4**	**235 (16.9)**
I am not allowed to report adverse events.	1.94	0.694	2	1–4	219 (15.7)
Adverse events cannot be avoided; therefore, there is no reason to report them.	2.13	0.745	2	1–4	355 (25.6)
Colleagues are not worried when adverse events occur.	2.06	0.728	2	1–4	292 (21.0)
**Approach to Reporting Adverse Events**	**2.27**	**0.52**	**2.2**	**1–4**	**433 (31.2)**
It is not my responsibility to report colleagues who are involved in an adverse event.	2.39	0.75	2	1–4	598 (43.1)
If those around you learn from adverse events, there is no need to report them.	2.21	0.711	2	1–4	425 (30.6)
There is no need to report minor adverse events.	2.28	0.711	2	1–4	492 (35.4)
Only rare adverse events should be reported.	2.22	0.721	2	1–4	441 (31.8)
Only adverse events from which lessons can be learned should be reported.	2.25	0.78	2	1–4	491 (35.4)
**Perceived Consequences of Adverse Event Reporting**	**2.48**	**0.47**	**2.4**	**1–4**	**689 (49.6)**
Reporting adverse events allows others to verify me.	2.7	0.672	3	1–4	933 (67.2)
The careers of staff who report adverse events suffer.	2.28	0.722	2	1–4	480 (34.5)
Adverse event reports cause a lot of trouble for me.	2.46	0.754	2	1–4	663 (47.8)
Adverse event reporting lets everyone know I made a mistake.	2.52	0.723	3	1–4	749 (54.0)
Adverse event reports encourage colleagues to gossip about my mistakes.	2.45	0.79	2	1–4	672 (48.4)
**Promoting a Supportive Culture for Adverse Event Reporting**	**2.54**	**0.62**	**2.5**	**1–4**	**940 (67.7)**
I receive encouragement from experienced colleagues to report adverse events.	2.56	0.715	3	1–4	780 (56.2)
The hospital’s adverse event monitoring unit would encourage staff to report errors.	2.53	0.747	3	1–4	744 (53.6)
**Organizational Procedures for Reporting Adverse Events**	**2.87**	**0.64**	**3**	**1–4**	**1147 (82.6)**
The hospital where I work has clear procedures for how to report adverse events.	2.88	0.726	3	1–4	1046 (75.4)
The hospital where I work has clear procedures for what adverse events should be reported.	2.85	0.711	3	1–4	1013 (72.9)

* Positive answers: for subscales—average value of all items ≥2.5; for separate items—sum of “agree” and “strongly agree” cases.

**Table 4:** Relationship between domains of clinical practice environment and nurses reporting adverse events.

RPPE Subscale	Reported an Adverse Event N (%)	Didn’t Report an Adverse Event N (%)	χ^2^, *p*-Value
Clinical Practice: Leadership and Autonomy	502 (88.7)	707 (86.0)	2.148, 0.143
Control of the Practice	288 (50.9)	444 (54.0)	1.319, 0.251
Patient-related Communication	475 (83.9)	699 (85.0)	0.319, 0.572
Work in a Team	384 (67.8)	598 (72.7) *	3.897, 0.048
Dealing with Conflicts	426 (75.3)	619 (75.3)	0.000, 0.987
Workplace Internal Motivation	552 (97.5)	795 (96.7)	0.769, 0.380

* Statistically significant difference comparing “Didn’t report an adverse event” group with “Reported an adverse event” group.

**Table 5:** Relationship between domains of the clinical practice environment and nurses who will report adverse events in the future.

RPPE Subscale	Will Report Adverse Events in the Future N (%)	Will Not Report Adverse Events in the Future N (%)	χ^2^, *p*-Value
Clinical Practice: Leadership and Autonomy	689 (89.0) **	520 (84.7)	5.708, 0.017
Control of the Practice	415 (53.6)	317 (51.6)	0.543, 0.461
Patient-related Communication	669 (86.4) **	505 (82.2)	4.602, 0.032
Work in a Team	567 (73.3) **	415 (67.6)	5.312, 0.021
Dealing with Conflicts	603 (77.9) **	442 (72.0)	6.450, 0.011
Workplace Internal Motivation	758 (97.9) **	589 (95.9)	4.799, 0.028

** Statistically significant difference comparing “Will report adverse events in the future” group with “Will not report adverse events in the future” group.

## Data Availability

The data presented in this study are available on request.
